# Supplementary figures and images for: Interactions between microbiota and uterine corpus endometrial cancer: A bioinformatic investigation of potential immunotherapy
Source: PLoS One. 2024 Oct 30;19(10):e0312590. doi: 10.1371/journal.pone.0312590 (PMC11524446; doi:10.1371/journal.pone.0312590)

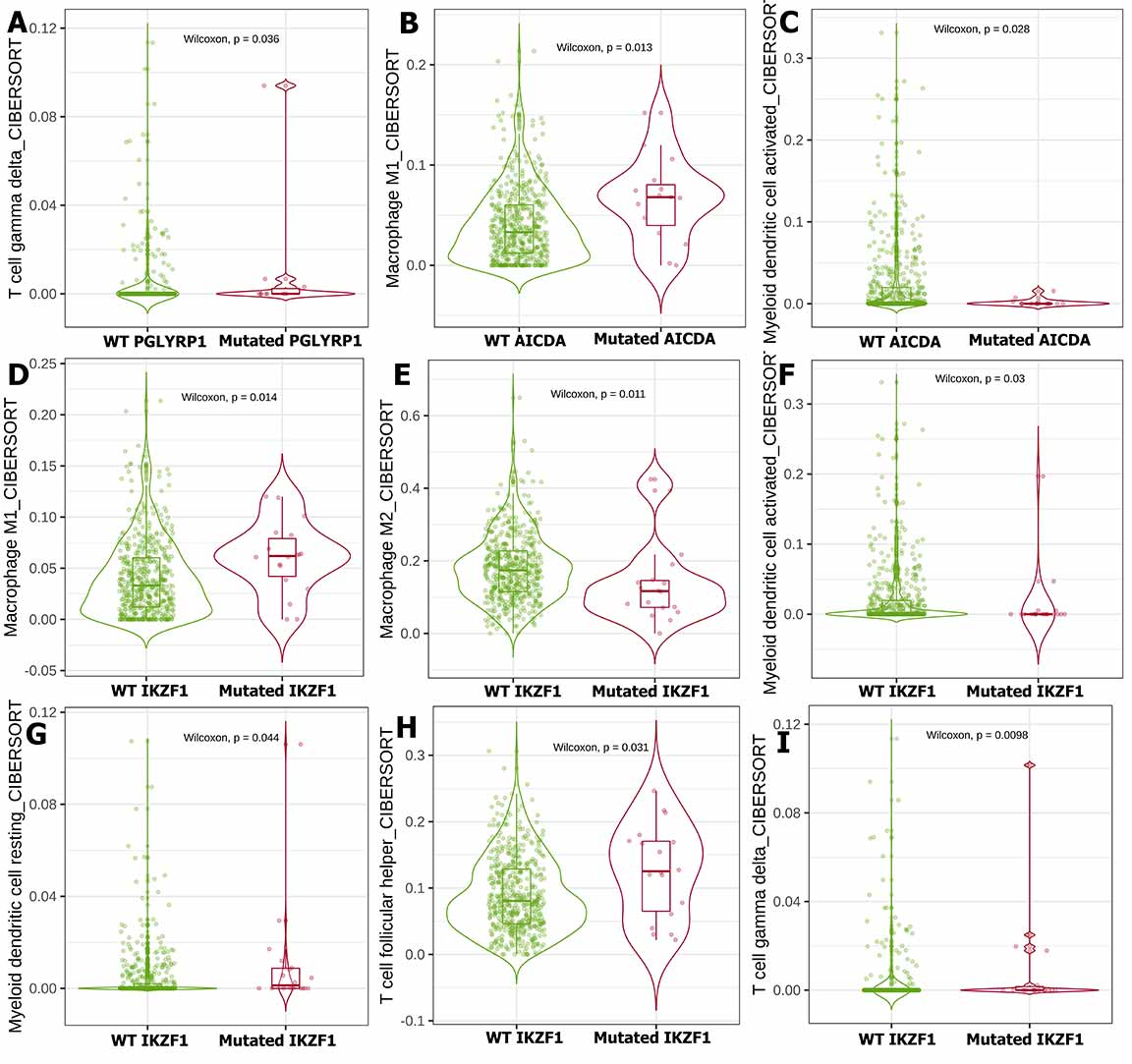

Supplement: S1 Fig — The PGLYRP1 status affect T cell gamma delta infiltration in UCEC patients (A). Whereas AICDA status affect M1 macrophage infiltration (B) and myeloid dendritic cell activation (C). M1 macrophages (D), M2 macrophages, myeloid dendritic cell active (E), myeloid dendritic cell resting (F), T cell follicular helper (H), and T cell gamma delta (I) infiltration are all affected by IKZF1 status. (TIF) [file pone.0312590.s001.tif]

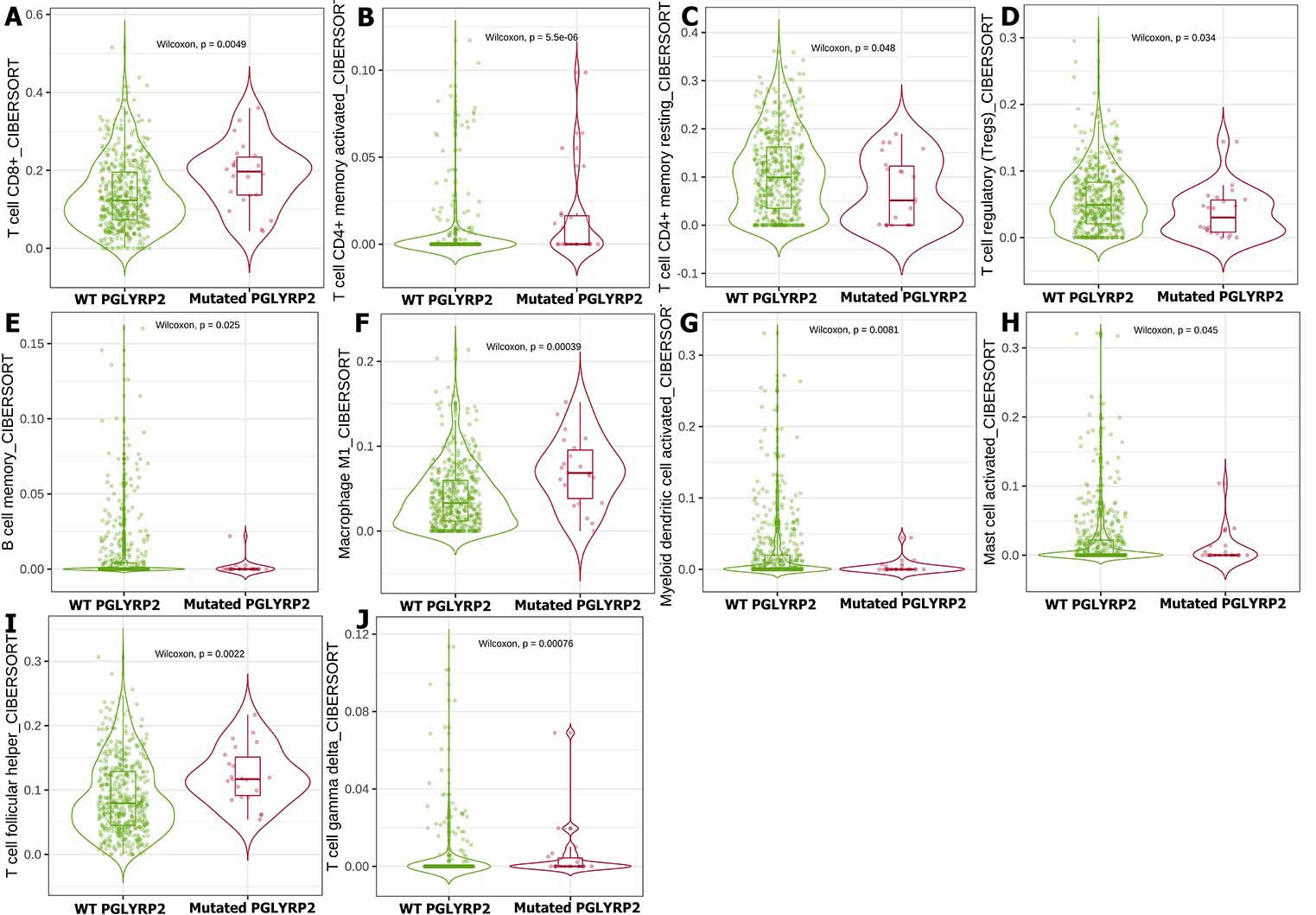

Supplement: S2 Fig — PGLYRP1 status orchestrates T cell CD8+ (A), CD4+ memory activated (B), CD4+ memory resting (C), Tregs (D), B cell memory (E), M1 macrophages (F), Myeloid dendritic cell activated (G), mast cell activated (H), T cell follicular helper (I), and T cell gamma delta (J) invasion in UCEC patients. (TIF) [file pone.0312590.s002.tif]

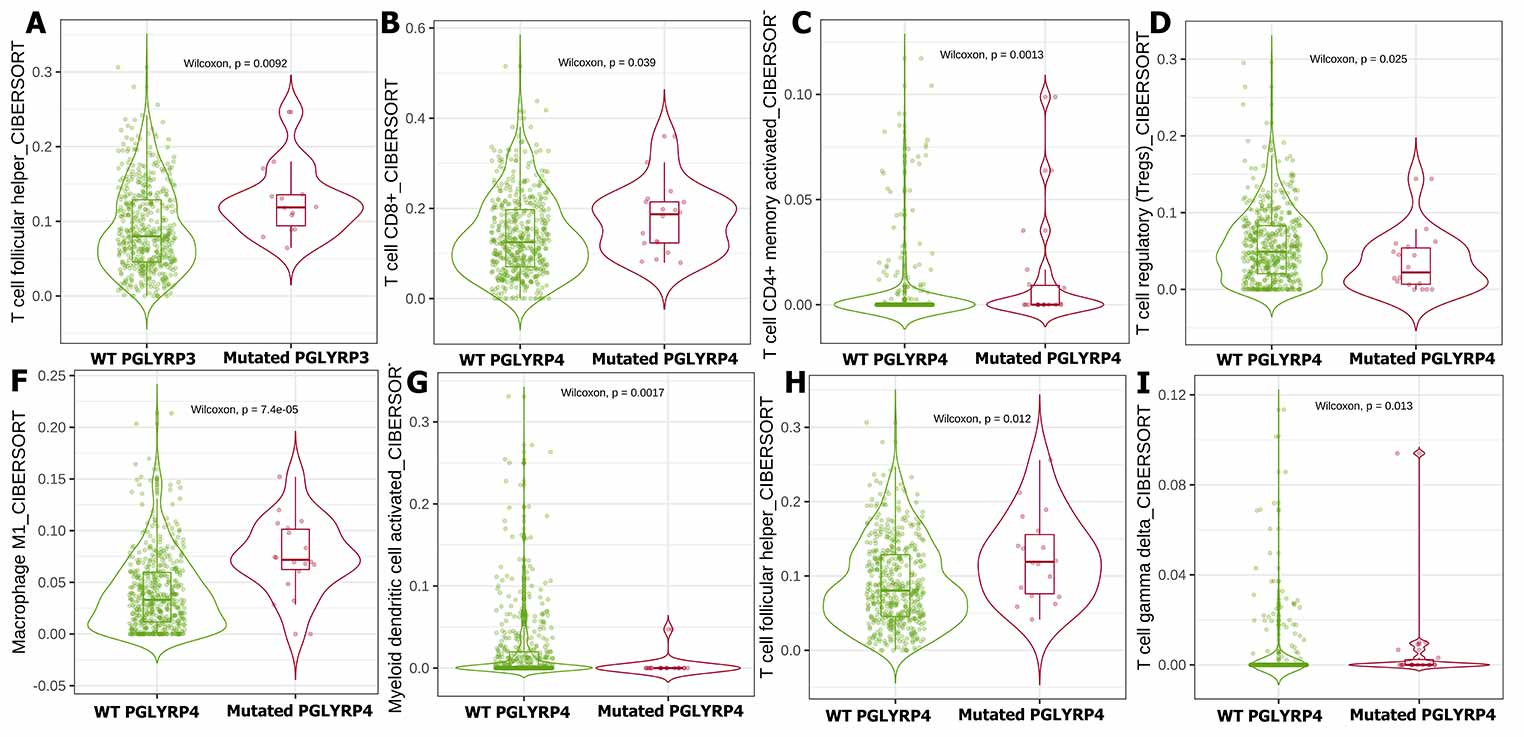

Supplement: S3 Fig — The PGLYRP3 status influences cell T-cell follicular helper infiltration in UCEC patients (A). In UCEC patients, PGLYRP4 status affects T cell CD8+ (B), CD4+ memory activated (C), Tregs (D), M1 macrophages (F), myeloid dendritic cell activated (G), T cell follicular helper (H), and T cell gamma delta (I) invasion. (TIF) [file pone.0312590.s003.tif]

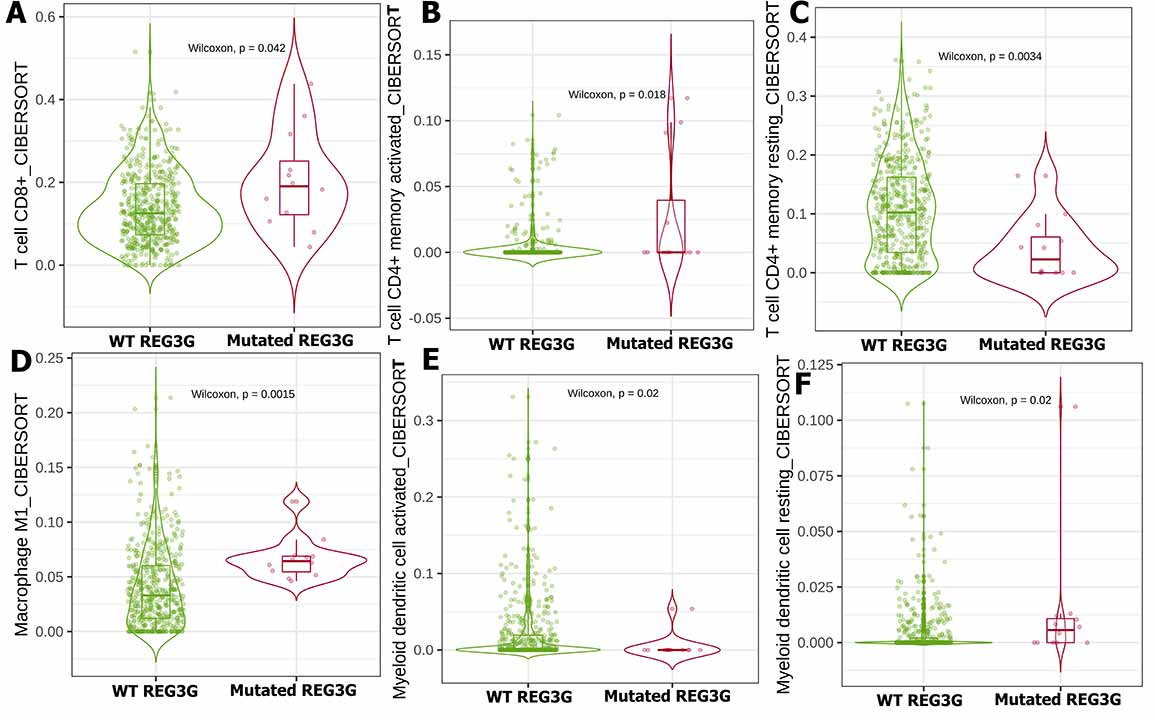

Supplement: S4 Fig — T cell CD8+ (A), CD4+ memory activated (B), CD4+ memory resting (C), M1 macrophages (D), Myeloid dendritic cell active (E), and myeloid dendritic cell resting (F) invasion is controlled by REG3G status in UCEC patients. (TIF) [file pone.0312590.s004.tif]

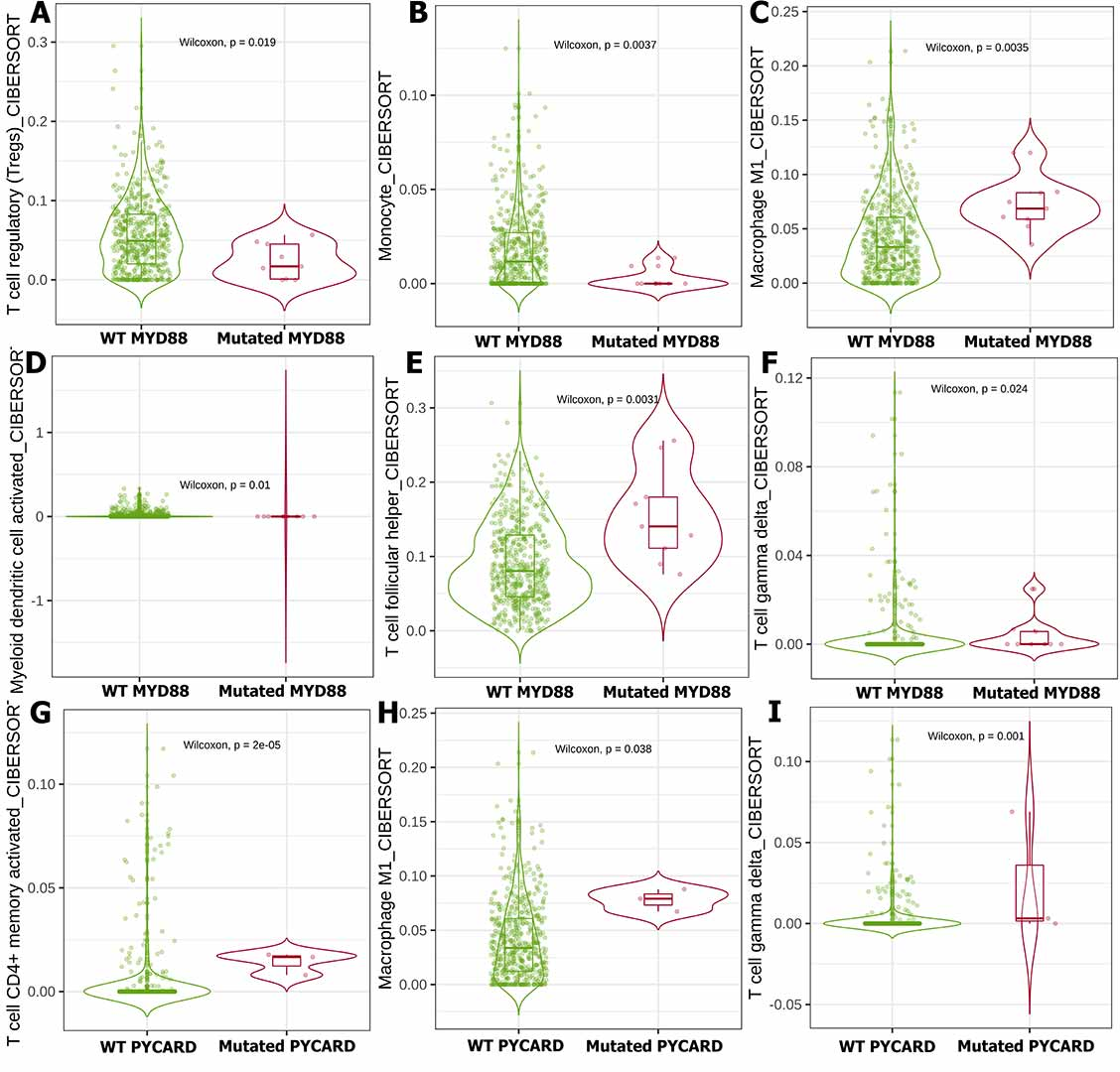

Supplement: S5 Fig — Tregs (A), monocytes (B), M1 macrophages (C), myeloid dendritic cell activated (D), T cell follicular helper (E), and T cell gamma delta (F) infiltration in UCEC patients are influenced by MYD88 status. PYCARD status influences CD4+ memory activated (G), M1 macrophage (H), and T cell gamma delta (I) invasion in UCEC patients. (TIF) [file pone.0312590.s005.tif]
